# Improving Transition of Care for Pediatric Patients With Chronic Kidney Disease: A Pilot Project

**DOI:** 10.7759/cureus.63367

**Published:** 2024-06-28

**Authors:** Melvin Chan, Sarah Young, Melisha Hanna

**Affiliations:** 1 Pediatric Nephrology, Children’s Hospital Colorado, Aurora, USA; 2 Nephrology, University of Colorado Anschutz Medical Campus, Aurora, USA

**Keywords:** remote teaching, self-management counseling, adolescent and young adult (aya), health - knowledge, transition to adult health care, patient-centered approach, chronic kidney disease (ckd)

## Abstract

Introduction

Transition is the process of preparing an adolescent or young adult for the adult model of care. Poor transitions have been linked to increased medical utilization and poorer kidney outcomes. There are limited studies evaluating predictors of transition readiness or interventions in pediatric patients with chronic kidney disease (CKD).

Methods

We enrolled 42 non-dialysis, non-transplant patients with CKD stage 2 or higher and 14 years and older receiving care in our pediatric nephrology clinic. Data collected included demographics, clinical information, and transition readiness as measured by the Transition Readiness Assessment Questionnaire (TRAQ). Patients were provided with a structured, remote curriculum with resources that addressed areas of need. Patients were followed every three to six months. Repeat TRAQ questionnaires were administered six months after enrollment.

Results

Our study found that younger age and male gender were risk factors for poor transition. Age was consistently a positive predictor of higher TRAQ scores in the medication, appointment, and total score domains (p < 0.05). Male gender was a risk factor for lower TRAQ scores in the appointment and communication domains (p < 0.05). Additionally, our curriculum was effective at improving scores across all TRAQ domains, with an average increase of about 25% in six months. There was no difference in patients who had a three-month follow-up as compared to a six-month follow-up (p > 0.05).

Conclusion

Our study finds that younger age and male gender are risk factors for poor transition. Additionally, a structured, remote curriculum is effective at improving transition readiness.

## Introduction

Medical treatment improvements have resulted in as much as 90% of children with chronic illnesses surviving into adulthood, all of whom will need an adult provider [1]. Poor transition can result in increased medical utilization and a higher risk of graft rejections in kidney transplant recipients [1,2]. A recent report from the National Survey of Children’s Health in 2019 showed that only 23% of children with chronic illness received a formal transition education, which has been shown to improve adherence to care, disease-specific measures, quality of life, satisfaction with care, and healthcare utilization [1,3,4].

Transitioning adolescents and young adults (AYA) with childhood-onset conditions to adult medical care is challenging for multiple reasons. Adolescence is an important period of multiple changes, specifically as it relates to identity, education, and sexuality [5]. Having a chronic condition, such as sickle cell disease or chronic kidney disease (CKD), can complicate this developmental period [5]. Studies among AYA with chronic health conditions have shown lower self-esteem and higher rates of depressive symptoms and suicidal thoughts [6,7]. Therefore, a multidisciplinary team is required to help AYA through this developmental period.

From a provider perspective, there are logistical and financial barriers. At our institution, patients come from seven states, making it difficult to coordinate fragmented care. Additionally, limited clinic time is focused on medical care rather than educating patients about transition. Dedicated transition clinics are resource-intensive, requiring multidisciplinary personnel, including social workers, navigators, providers, and pharmacists. Lastly, payment systems do not reimburse this complex care coordination; multiple studies have highlighted the importance of navigators for successful transitions [5,8,9].

Because of these challenges, there are no standard best practices for developing a transition program [5]. Previous interventions include transition camps, mentorship programs, and transition coordinators, with most studies finding positive qualitative outcomes, like increased motivation [10]. Of the studies evaluating quantitative outcomes, most come from multidisciplinary transition clinics involving AYA solid organ transplant recipients; these clinics improve follow-up with adult providers, medication adherence, and renal function [11,12]. In our review of the literature, none have evaluated improving transition readiness through a structured curriculum and correlating readiness with outcomes.

The goal of this pilot project is to evaluate the effectiveness of a structured education curriculum for AYA with CKD. This paper will describe predictors of transition readiness, a remote transition curriculum and its impact on transition readiness, and feedback from participants and their families. A follow-up study will evaluate the outcomes, such as medical utilization and clinical parameters, for patients who transfer to an adult provider.

## Materials and methods

We conducted a prospective pilot project at Children’s Hospital Colorado in Aurora, a freestanding children’s hospital with over 600 beds across four campuses and a quaternary referral center in the western United States. The study included all follow-up patients aged 14 and older with CKD stage 2 or higher. Exclusion criteria included those with intellectual disabilities, who had a transplant, or who were on dialysis, as these patients had different transition processes. Eligible patients were recruited through the patient portal or by telephone call. At our center, over 90% of patients used MyChart to communicate with their healthcare team (Epic, Verona, United States). Interested patients were sent a message with the Transition Readiness Assessment Questionnaire (TRAQ) and received postcard consent through the patient portal or email. The Colorado Multiple Institutional Review Board approved this study (approval number 21-4622).

Predictors

Patient demographic data was collected from the electronic health record (EHR), including age, gender, and type of insurance. Additionally, the following information on medical utilization in the prior year was gathered: the number of emergency room visits, hospitalizations, average length of stay per hospitalization, and number of specialty appointments attended at our institution. Finally, the patient’s CKD staging was recorded as defined by the Kidney Disease: Improving Global Outcomes [13]. Patients who had stage 1 or 2 CKD were categorized as having mild kidney disease, whereas the other stages were categorized as being moderate to severe.

Transition readiness

The primary outcome was transition readiness, as measured by TRAQ. This questionnaire is a well-validated, 20-item patient-report survey that has four key domains assessing confidence in medication management, appointment keeping, healthcare knowledge, and communication (Appendix A) [14].

**Figure 1:**
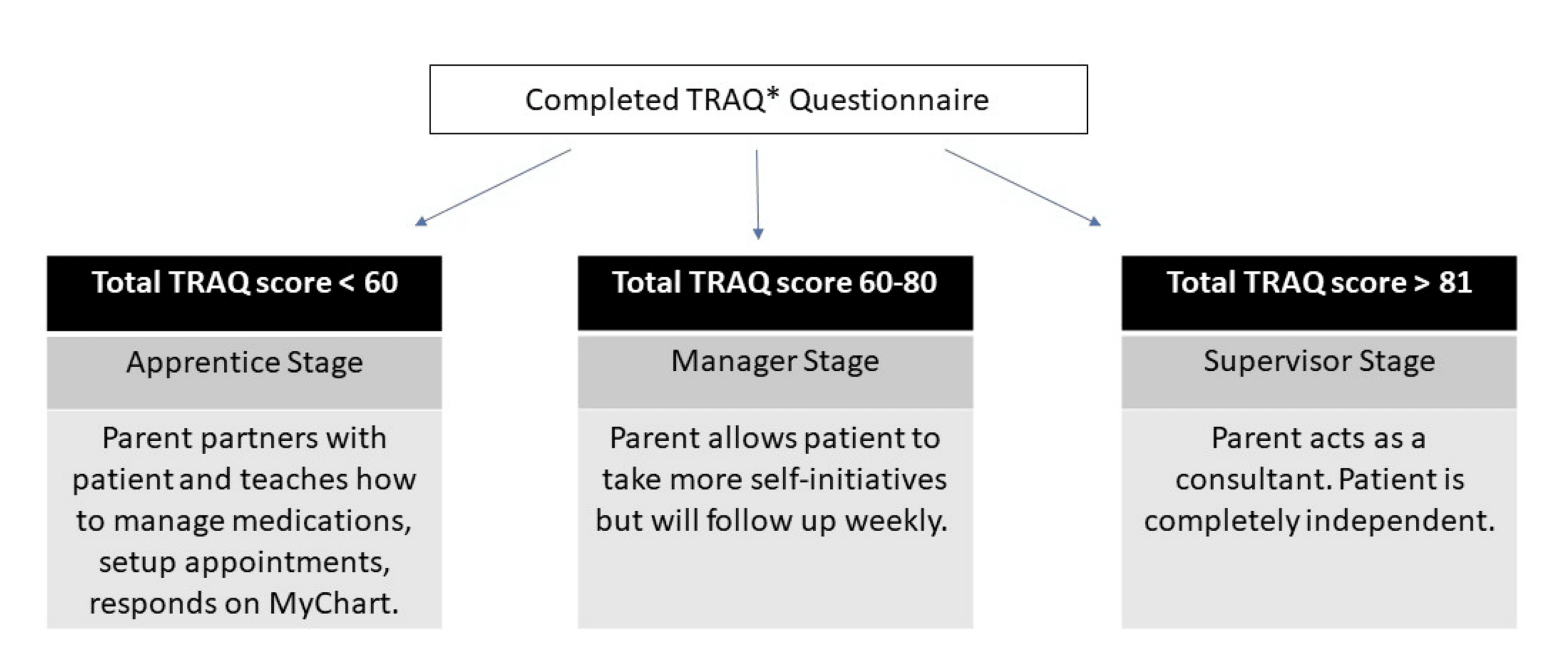
Staging based on TRAQ* score Based on the total TRAQ score, patients were placed in different stages. The “apprentice” stage corresponded to patients scoring less than 60 points and required the most parental support. Scores of 60-80 points corresponded to the “manager” stage needing less supervision. Patients scoring over 80 points were in the “supervisor” stage and were the most independent. *TRAQ, Transition Readiness Assessment Questionnaire

All patients completed the survey before enrollment. After enrollment, patients were placed into stages based on their total scores and encouraged to collaborate with their parents, as shown in Figure 1.

If patients scored less than 90% of the total domain score, handouts were sent via the patient portal. Patients struggling with medication knowledge or skills were given a tip sheet (Appendix B). For patients who needed help with healthcare knowledge, they were provided a kidney passport, a handout on CKD basics, and commonly used CKD medications (Appendices C-E). Patients needing help with appointments and communications were encouraged to get access to their own patient portal in addition to their parent’s account.

After these initial interventions, patients were provided two follow-up options: education only (EO) or education and coaching (EC). In the EO cohort, patients had a follow-up every six months, whereas those in the other cohort had one every three months. These follow-up sessions were conducted via the patient portal and a telephone call. During the follow-up, patients were commended for their progress and coached in deficient areas, and key elements of the handouts were reinforced. During these follow-ups, read receipt notifications were used to track adherence to handouts. TRAQ was readministered every six months, regardless of the follow-up option. Failure to complete the follow-up question was characterized as a lost to follow-up.

Feedback

During TRAQ readministration at six months, patients were asked the following: (1) How is the program going?; (2) What are the strengths of this program?; (3) What are the weaknesses of this program?; (4) How can we improve these weaknesses?

Statistical analysis

Patient characteristics were summarized using medians (IQRs) for continuous variables and counts (percentages) for categorical data. Statistical comparisons of patients who did and did not complete their follow-up surveys were performed using the Mann-Whitney U test for continuous variables and the Pearson chi-square test for categorical data. Bivariate associations between predictors and TRAQ scores were analyzed using linear regression. Multivariable linear regression models, based on variables with p < 0.05 in bivariate analysis, were used to account for potential confounding variables. A Wilcoxon signed-rank test was used to compare initial and six-month follow-up TRAQ scores. A repeated measures ANOVA was used to evaluate the effects of the EC and EO cohorts. Analysis was performed using IBM SPSS Statistics for Windows, Version 29.0 (Released 2022; IBM Corp., Armonk, NY, USA).

## Results

Out of a total of 151 eligible patients, 42 patients were enrolled in this pilot project, with a participation rate of 27.8%. Table 1 describes the clinical characteristics of these patients.

**Table 1:** Clinical characteristics of participants A single hyphen denotes a blank cell. CKD, chronic kidney disease; NA, no available data; TRAQ, Transition Readiness Assessment Questionnaire

Variables	Eligible patients (n = 151)			Full enrolled cohort based on follow-up survey status (n = 42)		
Median (IQR)/count (%)	Unenrolled cohort (n = 109)	Enrolled cohort (n = 42)	p-value	No follow-up survey (n = 28)	Had follow-up survey (n = 14)	p-value
Age (years)	16 (15-18)	16 (15-17)	0.227	16 (15-17)	16 (15-17)	0.65
Gender	-		0.601	-		0.166
Male	67 (62%)	24 (57%)	-	18 (64%)	8 (57%)	-
Female	42 (38%)	18 (43%)		10 (36%)	6 (43%)	
Race	-		0.971	-		0.836
Caucasian	57 (53%)	22 (52%)	-	15 (54%)	7 (50%)	-
Non-Caucasian	52 (47%)	20 (48%)		13 (46%)	7 (50%)	
Insurance	-		0.628	-		0.199
Private insurance	57 (53%)	24 (57%)	-	15 (54%)	6 (43%)	-
Public insurance/no insurance	52 (47%)	18 (43%)		13 (46%)	8 (57%)	
Kidney disease severity	-		0.553	-		0.667
Mild	72 (66%)	30 (71%)	-	20 (71%)	11 (79%)	-
Moderate/severe	27 (34%)	12 (29%)		8 (29%)	3 (21%)	
Number of specialty clinics	NA	4 (3-9.5)	-	4 (2-9)	6.5 (3-11)	0.212
TRAQ initial scores	-					
Medications	NA	13.5	-	12.5	15.5	0.28
Appointment	NA	7.5		7	8.5	0.873
Healthcare knowledge	NA	19		17.5	20.5	0.583
Provider communication	NA	22		22	21.5	0.859
Total	NA	59.5		55	63.5	0.34

There were no differences between non-enrollees and enrollees, particularly in terms of age, race, gender, insurance type, and CKD severity. Among non-enrollees, 22% reported interest in participating but did not return a survey. The median age of the full cohort of participants was 16 years old. Approximately 70% of the patients had mild CKD. In the prior year, the median specialty clinic attendance was four visits. The median total TRAQ score was 59.5 out of a total of 100 points. When this cohort was divided based on the completion of the follow-up survey, there were no statistical differences between cohorts.

Predictors

Table 2 depicts the association between predictors and baseline TRAQ scores. Age was consistently a positive predictor of higher TRAQ scores in the medication, appointment, and total score domains (p < 0.05). Male gender was a risk factor for lower TRAQ scores in the appointment and communication domains (p < 0.05). After adjusting for potential confounding factors, age was still a positive factor in the appointment domain. None of the other variables correlated with TRAQ scores.

**Table 2:** Predictors of transition readiness A single hyphen denotes a blank cell. * A positive β indicates a protective predictor, whereas a negative β indicates a risk factor. ** p < 0.05 CKD, chronic kidney disease; TRAQ, Transition Readiness Assessment Questionnaire

TRAQ section/variables	Bivariate analysis		Multivariable analysis	
	β*	p-value	β*	p-value
TRAQ: Medications	-			
Age (years)	1.24	0.039**	-	
Male gender	-1.44	0.464		
Non-Caucasian	0.62	0.751		
Private insurance	1.17	0.546		
Number of emergency room visits	-0.52	0.336		
Number of hospitalizations	-0.2	0.845		
Average length of stay	-0.29	0.38		
Number of subspecialty clinic appointments	-0.14	0.371		
Moderate/severe CKD	-0.04	0.987		
TRAQ: Appointments	-			
Age (years)	1.16	0.010**	1.05	0.025**
Male gender	-2.13	0.043**	-1.12	0.167
Non-Caucasian	0.47	0.752	-	
Private insurance	-0.87	0.56		
Number of emergency room visits	-0.71	0.083		
Number of hospitalizations	-0.43	0.578		
Average length of stay	-0.18	0.485		
Number of subspecialty clinic appointments	-0.21	0.081		
Moderate/severe CKD	-1.78	0.297		
TRAQ: Healthcare knowledge	-			
Age (years)	0.16	0.92	-	
Male gender	-7.65	0.131		
Non-Caucasian	6.46	0.198		
Private insurance	-4.8	0.342		
Number of emergency room visits	-0.51	0.718		
Number of hospitalizations	-1.18	0.655		
Average length of stay	-0.42	0.628		
Number of subspecialty clinic appointments	-0.33	0.436		
Moderate/severe CKD	-2.56	0.662		
TRAQ: Provider communication	-			
Age (years)	0.07	0.965	-	
Male gender	-8.65	0.042**		
Non-Caucasian	4.82	0.336		
Private insurance	-2.99	0.553		
Number of emergency room visits	-0.13	0.929		
Number of hospitalizations	-0.4	0.879		
Average length of stay	-0.2	0.814		
Number of subspecialty clinic appointments	-0.23	0.578		
Moderate/severe CKD	-1.78	0.76		
TRAQ: Total score	-			
Age (years)	7.03	0.002**	-	
Male gender	-6.93	0.374		
Non-Caucasian	-0.51	0.948		
Private insurance	3.07	0.692		
Number of emergency room visits	-1.92	0.372		
Number of hospitalizations	1.01	0.802		
Average length of stay	-0.36	0.782		
Number of subspecialty clinic appointments	-0.33	0.606		
Moderate/severe CKD	-4.59	0.607		

Transition readiness

**Figure 2:**
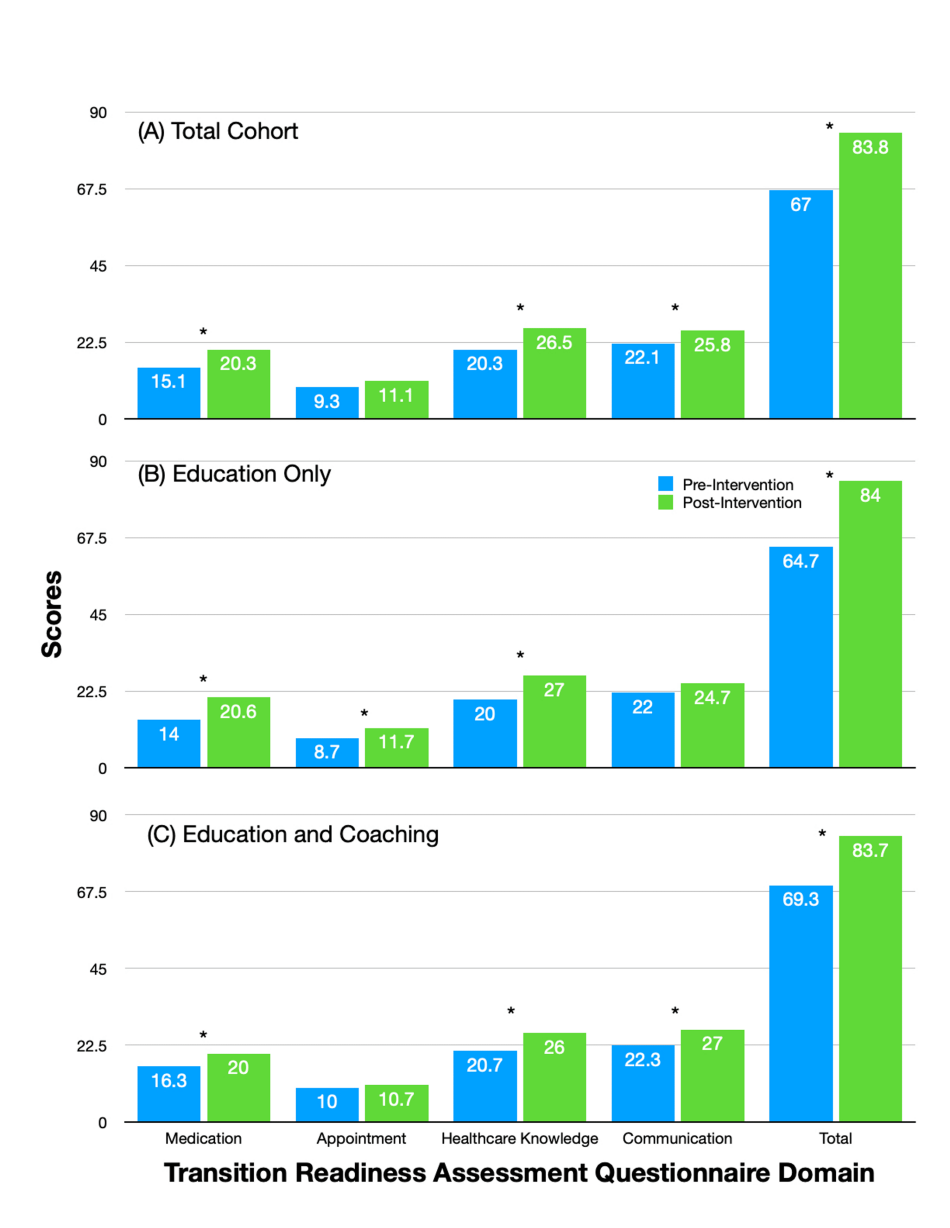
Changes in TRAQ scores over six months * p < 0.05 Baseline scores are shown in blue. Handouts are given to all patients, addressing areas of deficit. A repeat evaluation of transition readiness is done six months later, with scores shown in green. The total cohort is shown in panel A. The total cohort is further broken down based on follow-up options: EO (panel B) and EC (panel C). In the EO option, patients are followed up at six months during reevaluation. In the EC option, patients are followed up at three months with a telephone call and again at six months during reevaluation. EC, education and coaching; EO, education only; TRAQ, Transition Readiness Assessment Questionnaire

A total of 27 of the 42 patients (64%) were eligible for the six-month follow-up survey. The remainder of the participants were ineligible, as they had been in the program for less than six months. Four patients were transferred to an adult provider, and one had passed away, leaving 22 patients in the follow-up analysis. These patients were noted to have used the handouts based on their receipt notification. Altogether, we received 14 follow-up surveys, with a return rate of 63.6%; nine patients were in the EO cohort and five were in the EC cohort. Across both cohorts, scores were significantly improved from baseline across all domains except appointments. In the total cohort, the total score improved by 25% in six months (Figure 2A). One-year TRAQ scores were not compared, as only three patients had available data. Further analysis showed that the EO cohort had significant improvements in all domains except communication; the EC cohort also showed significant improvements except in appointments (Figure 2B, 2C). When the relative improvements were compared in the EO and EC cohorts, we were not able to find a significant difference (p > 0.05).

Feedback

All patients with completed follow-up surveys gave positive feedback. Major strengths included a structured curriculum, available resources, and routine follow-ups. Almost all parents reported that the project helped them realize the importance of transition planning. One commonly reported weakness was the lack of interactive material. Suggestions included the use of videos or animations and the employment of transition coordinators.

## Discussion

This study signifies that demographic factors need to be considered in determining transition readiness in AYA with CKD. In this study, we have two significant findings: age and male gender. Older patients have higher TRAQ scores than younger patients. This result has been reported in a previous CKD study [15]. Additionally, the male gender is a risk factor for lower TRAQ scores. The female gender is possibly protective because of its greater developmental maturity [16]. However, the role of gender still remains mixed; some studies have found male gender to be a risk factor and others protective [17,18].

Our study also demonstrates the effectiveness of a remote transition curriculum. Across all TRAQ domains except in appointments, there have been score improvements over the past six months. When the total cohort is divided based on follow-up, we are not able to find more robust score improvements. In the EC cohort, there are only five patients, and these participants have higher baseline scores than the EO cohort. Such a difference and a small sample size can explain why we have not seen an additive improvement with closer follow-up. Regardless, these results imply that time-intensive interventions may not always be more effective. Feedback has been positive, with most patients citing the benefits of a structured curriculum. Additionally, caregivers have a greater appreciation for transition planning. Taken together, our data show that transition readiness can be improved with regular screening and a simple, structured curriculum; a resource-intense multidisciplinary clinic is not always necessary to help improve readiness [11,12].

There are several limitations to our study. First, this study uses a convenience sample and only has a participation rate of 27.8%. There can be a selection bias where only highly motivated patients are enrolled. Such a bias can overestimate the improvement in scores. Additionally, the sample size of follow-up surveys is small, making it difficult to draw more significant conclusions. Furthermore, there is only a six-month follow-up period. It can be helpful to see if scores are sustained or continue to improve. Our study does have three one-year follow-up surveys, and scores continue to improve. Finally, it is difficult to tease apart whether scores show improvement from the educational curriculum, the survey, or both. A mother, after completing the initial survey, once mentioned, “I did not realize how much I was doing for my son. It just became a habit.”

There are several future directions for this project. We hope to evaluate the effectiveness of implementing this project with males younger than 14 years of age. Our hypothesis is that males need more time in a structured program to improve their TRAQ scores. Additionally, we wish to evaluate the curriculum’s effectiveness with more participants. Next, we want to evaluate whether improved TRAQ scores at the time of transfer translate to less medical utilization, improved adherence, and less progression of kidney disease. Finally, we are working with the information technology team to automate this process using artificial intelligence [19]. Specifically, we are creating a program to have the EHR identify eligible patients and automatically send the transition surveys through the patient portal; additionally, the EHR will provide quick response codes that link to the appropriate resources based on survey responses.

## Conclusions

Preparing for the pediatric-to-adult transition is crucial for limiting unnecessary medical utilization and poor longitudinal kidney outcomes. Unfortunately, there is scarce data on risk factors and best practices for improving transition readiness in patients with CKD. Similar to other subspecialty studies, our study adds to a growing body of literature that has found younger age and male gender to be risk factors for transition readiness. Additionally, our study shows that a structured, remote curriculum can improve transition readiness over a six-month period for pediatric patients in a CKD clinic.

## Figures and Tables

**Figure 3:**
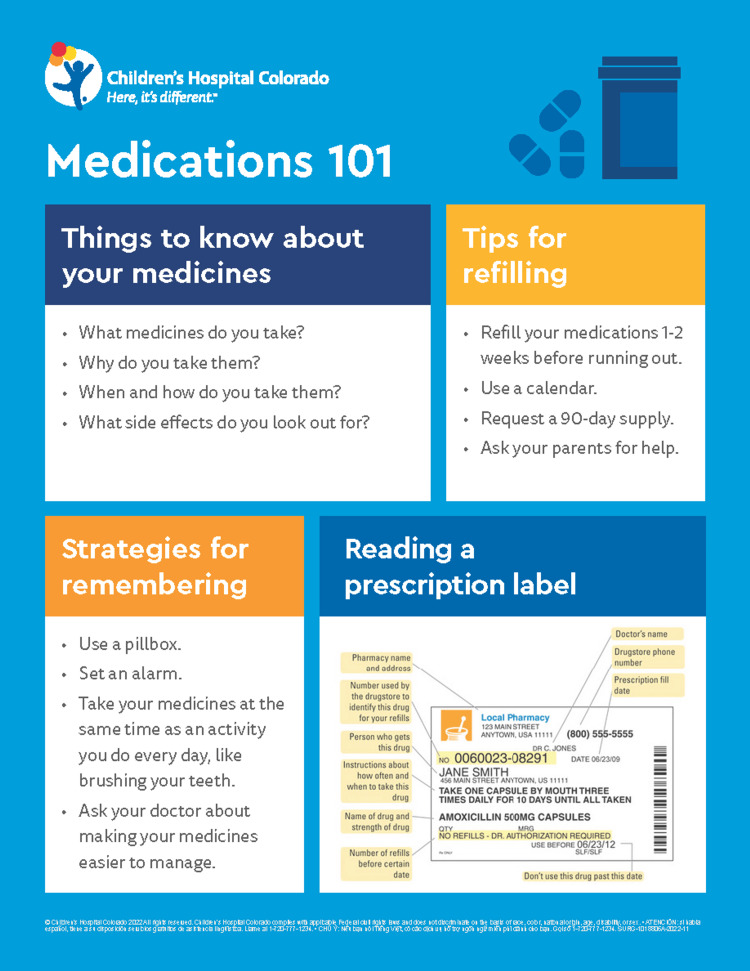
Medication Handout This handout provides tips for refilling medicines, strategies for remembering to take medicines regularly, and a tutorial in reading a prescription label.

**Figure 4:**
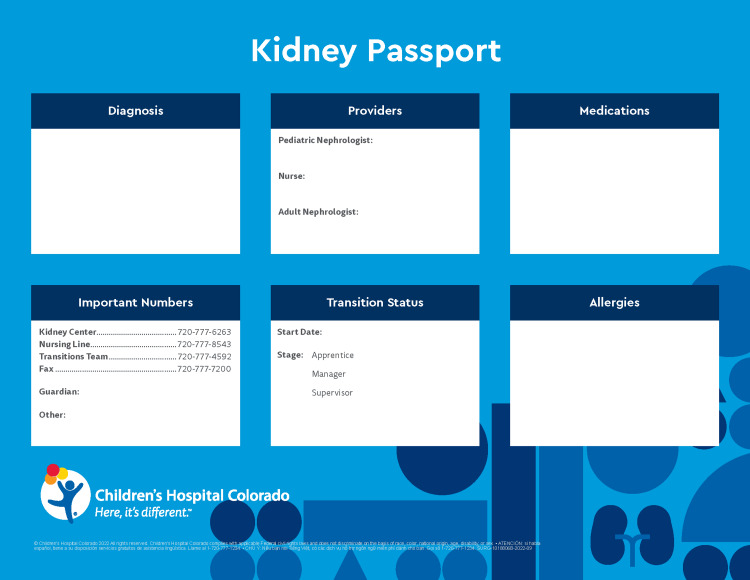
Kidney Passport This handout is a method to help patients learn about their diagnosis, providers, medications, and allergies. Additionally, it provides important clinic phone numbers.

**Figure 5:**
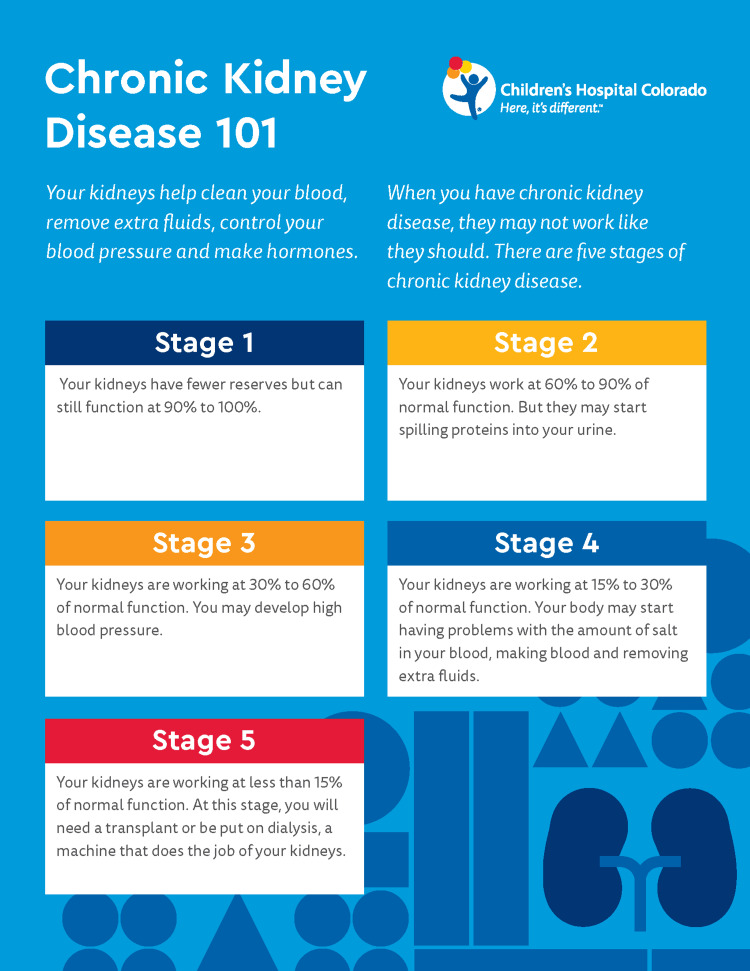
Chronic Kidney Disease Basics This handout provides basic information on chronic kidney disease.

**Figure 6:**
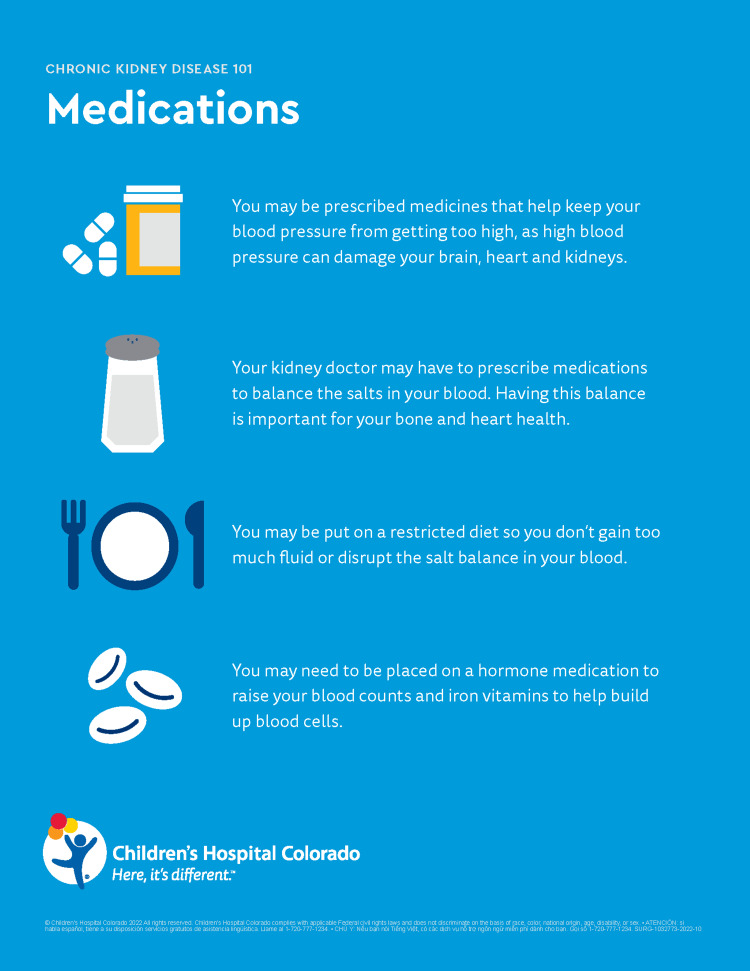
Commonly Used Medications in Chronic Kidney Disease This handout provides information on basic medicines or dietary changes used for patients with chronic kidney disease.

## Appendices

Appendix A

**Table 3:** The Transition Readiness Assessment Questionnaire Single hyphen: Denotes blank cell

Domains	Questions
Managing Medications	-
-	Do you explain any medications (name and dose) you are taking to healthcare providers?
-	Do you fill a prescription if need to?
-	Do you reorder medications before they run out?
-	Do you know what to do if you are having a bad reaction to your medications?
-	Do you speak with the pharmacist about drug interactions or other concerns related to your medications?
Appointment Keeping	-
-	Do you call the doctor’s office to make an appointment?
-	Do you keep a calendar or list of medical and other appointments?
-	Do you arrange for your ride to medical appointments?
Managing Health Issues	
-	Do you explain your medical history to your healthcare providers (including past surgeries, allergies, medications)?
-	Do you fill out the medical form, including a list of your allergies?
-	Do you make or help make medical decisions pertaining to your health?
-	Do you tell the doctor or nurse whether you followed their advice or recommendations?
-	Do you call the doctor about unusual changes to your health (For example: Allergic reactions)?
-	Do you follow-up on any referral for tests, check-ups, or labs?
Talking with Providers	
-	Do you tell the doctor or nurse what you are feeling?
-	Do you answer questions that you are asked by the doctor, nurse, or clinic staff?
-	Do you ask questions of your nurse or doctor about your health or health care?
-	Do you ask your doctor or nurse to explain things more clearly if you do not understand their instructions to you?
-	Do you attend your medical appointments or part of your appointment by yourself?
-	Do you contact the doctor when you have a health concern?

Appendix B

Appendix C

Appendix D

Appendix E
